# Novel Combined Antioxidant Strategy against Hypertension, Acute Myocardial Infarction and Postoperative Atrial Fibrillation

**DOI:** 10.3390/biomedicines9060620

**Published:** 2021-05-30

**Authors:** Ramón Rodrigo, Jaime González-Montero, Camilo G. Sotomayor

**Affiliations:** 1 Molecular and Clinical Pharmacology Program, Institute of Biomedical Sciences, Faculty of Medicine, University of Chile, CP 8380453 Santiago, Chile; rrodrigo@med.uchile.cl; 2 Basic and Clinical Oncology Department, Faculty of Medicine, University of Chile, CP 8380453 Santiago, Chile; jagonzalez@ug.uchile.cl; 3 Clinical Hospital University of Chile, University of Chile, CP 8380453 Santiago, Chile

**Keywords:** antioxidants, oxidative stress, ischemia, reperfusion, hypertension, acute myocardial infarction, postoperative atrial fibrillation

## Abstract

Reactive oxygen species (ROS) play a physiological role in the modulation of several functions of the vascular wall; however, increased ROS have detrimental effects. Hence, oxidative stress has pathophysiological impacts on the control of the vascular tone and cardiac functions. Recent experimental studies reported the involvement of increased ROS in the mechanism of hypertension, as this disorder associates with increased production of pro-oxidants and decreased bioavailability of antioxidants. In addition, increased ROS exposure is found in ischemia-reperfusion, occurring in acute myocardial infarction and cardiac surgery with extracorporeal circulation, among other settings. Although these effects cause major heart damage, at present, there is no available treatment. Therefore, it should be expected that antioxidants counteract the oxidative processes, thereby being suitable against cardiovascular disease. Nevertheless, although numerous experimental studies agree with this notion, interventional trials have provided mixed results. A better knowledge of ROS modulation and their specific interaction with the molecular targets should contribute to the development of novel multitarget antioxidant effective therapeutic strategies. The complex multifactorial nature of hypertension, acute myocardial infarction, and postoperative atrial fibrillation needs a multitarget antioxidant strategy, which may give rise to additive or synergic protective effects to achieve optimal cardioprotection.

## 1. Introduction

Cardiovascular diseases are a group of heterogeneous heart and circulatory disorders, asymptomatic for a long time yet evolving throughout life. Both ethnic and geographic factors are involved in its prevalence, together with risk factors such as smoking habits, lack of physical activity, increased body mass index, hypertension, dyslipidemia, and diabetes mellitus. Cardiovascular diseases are the first cause of mortality in western countries. By 2030, it was estimated that 23.6 million people per year would die due to these diseases (WHO). Hypertension is a major independent and progressive risk factor for cardiovascular disease. It remains a leading cause of morbidity and mortality worldwide, causing 10.4 million deaths a year. Hypertension is the most important contributor to cardiovascular disease burden and associated premature death globally [1]. Although considerable effort has been devoted to study the pathophysiology of hypertension, the underlying mechanisms need to be further elucidated. It has been indicated by experimental evidence that reactive oxygen species (ROS) play an important role in the pathophysiology of hypertension [2]. Indeed, oxidative stress plays a central role in cardiovascular diseases. Accordingly, conditions associated with the occurrence of ischemia followed by reperfusion lead to the development of oxidative stress, such as acute myocardial infarction or cardiac surgery with extracorporeal circulation. Consequently, structural and functional remodeling, together with functional effects could cause severe damage, even reaching cell death and increase of infarct size in the case of acute myocardial infarction. Despite antioxidants are molecules able to counteract the effects of oxidative stress, experimental studies and clinical trials have not provided convincing data until now. This inconsistency could be since the fact that the biological and health effects of any given antioxidant depend on numerous factors, such as the chemical reactivity toward radicals or another target related to oxidative stress, absorption, and distribution in body tissue. In addition, diverse models, molecules, and dosage of administration makes it difficult to draw definitive conclusions. In fact, more studies are still lacking to support the bases for a rational antioxidant therapy.

At present, the available studies involving the relationship between oxidative stress and the therapy of cardiovascular diseases have been mainly focused on the administration of antioxidants as monotherapies; however, the results of clinical studies remain disappointing. Recently, based on the fact that the pathophysiological nature of ischemia-reperfusion cardiac injury seems to be multifactorial, it was suggested that it requires multitarget therapeutic strategies [3]. Accordingly, it should be expected that an association of antioxidant agents could lead to an improvement in the pharmacological response, based on the combination of additive and/or synergistic effects. Nevertheless, this paradigm has not been explored enough.

In the following sections of this narrative review, we will present an update of the relationship between oxidative stress and the pathophysiology of hypertension, acute myocardial infarction and postoperative atrial fibrillation, as clinical models targeted with antioxidant therapy according to the available data.

## 2. Oxidative Stress and Antioxidant Defense System

Oxidative stress represents a common pathway leading to pathophysiological cascades in cardiovascular diseases [4]. The mechanism against the challenge of oxidative stress is composed of antioxidant enzymes, and molecules aimed to counteract the deleterious effect of ROS and reactive nitrogen species (RNS) on biomolecules (e.g., proteins, DNA, and lipids). The activity of antioxidant enzymes is subjected to genomic regulation according to the cell redox signaling mediated by the transcription factor Nrf2 (nuclear factor erythroid 2-related factor 2) on the antioxidant response elements. The operation of this protective response occurs with a low increase of hydrogen peroxide concentration (1 to 10 nM), but higher ones (>100 nM) lead to the activation of the NFκB pathway [5], thereby resulting in deleterious cell effects. Under oxidative conditions leading to the development of oxidative stress, the endogenous system fails to keep normal redox homeostasis. Hence, supplementation with antioxidant molecules is necessary to scavenge the free radicals and other reactive molecules [6]. There is evidence from prospective cohort studies and clinical trials that there is a protective effect by plasma/dietary antioxidants against the risk of cardiovascular events [7]. However, studies in humans remain inconsistent according to the findings of clinical trials, and more studies are still lacking.

### 2.1. Pathophysiological Effects of Ischemia Followed by Reperfusion

The configuration of ischemia-reperfusion events is unavoidable in several clinical settings, such as acute myocardial infarction, organ transplantation, stroke, and cardiac surgery with extracorporeal circulation, among others.

#### 2.1.1. Ischemia

During the ischemia phase, the absence of oxygen activates anaerobic respiration, leading to intracellular accumulation of lactic acid and a drop in the production of adenosine triphosphate (ATP) and metabolic activity (Krebs cycle). The decrease in intracellular pH leads to Na^+^ influx through the Na^+^/H^+^ exchanger, and ATP depletion contributes to intracellular Na^+^ accumulation, thereby activating Na^+^/Ca^2+^ exchanger in the reverse direction, which results in cytosolic Ca^2+^ overload. This event in the cardiac tissue overwhelms the sarcoplasmic reticulum Ca^2+^ uptake through Ca^2+^-ATPase. Acidosis and low ATP levels reduce myocardial contractile activity. In turn, increased cytosolic Ca^2+^ levels can activate several enzymes, including xanthine dehydrogenase, further increasing oxidative stress.

#### 2.1.2. Reperfusion

During the first minutes of reperfusion, a burst of ROS occurs in accordance with several experiments demonstrating direct evidence of free radicals in isolated hearts and in vivo ischemia-reperfusion models. In the ischemic phase, xanthine oxidase (XO) activation and ATP catabolism to hypoxanthine occur, generating high ROS levels and uric acid when blood flow is restored. Cardiolipin peroxidation and cytochrome oxidase uncoupling in the ischemic period resulted in the inhibition of electron flux through mitochondrial electron transport chain, ATP depletion, and increased superoxide anion generation, which persists in the reperfusion phase, where the Krebs cycle is reactivated and high levels of tissue oxygen can lead to further increased ROS production [8]. In addition, in prolonged ischemia tetrahydrobiopterin (BH4), an endothelial NOS (eNOS) coupling factor, suffers oxidation to dihydrobiopterin, resulting in loss of eNOS enzyme affinity by the substrate L-arginine together with a shift in the generation of nitric oxide, a potent vasodilator, to superoxide anion instead during reperfusion.

**Figure 1:**
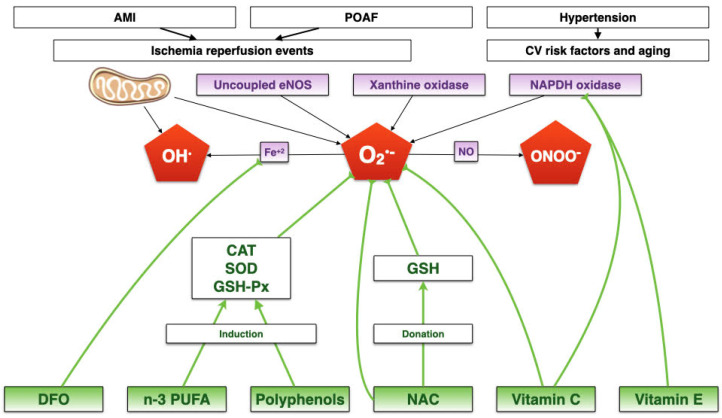
Integrative summary of how oxidative stress could be an underlying mechanism for postoperative atrial fibrillation, acute myocardial infarction, and hypertension. There are four main ROS sources, producing mainly superoxide anion. ROS production can be amplified through the interaction with nitric oxide and iron, to produce peroxynitrite and hydroxyl radical, respectively. CV: Cardiovascular. AMI: Acute myocardial infarction. NO: Nitric oxide. Fe^+3^: Free ferric iron. OH: Hydroxyl anion. CAT: Catalase. GSH-Px: Gluthathione peroxidase. SOD: Superoxide dismutase. n-3 PUFA: Polyunsatured fatty acids. DFO: Deferoxamine. NAC: N-acetylcysteine. GSH: Glutathione. POAF: Postoperative atrial fibrillation. ONOO-: Peroxynitrite. OH•: Hydroxyl radical. O2•-: superoxide anion. eNOS: Endothelial nitric oxide synthase.

The integrative mechanisms involved in these diseases are depicted in Figure 1.

## 3. Hypertension

Firstly, it is of interest to note that hypertension may be the most important contributor to cardiovascular disease burden [1]. Unfortunately, it remains a silent killer since about one-third of hypertensive patients are unaware of having this condition, thereby increasing the risk of stroke and heart disease, among other deleterious effects. 

The relationship between hypertension and oxidative stress arises from the modulation of the vasomotor tone at the level of the vascular wall. The imbalance of vasodilator and vasoconstrictor forces may be caused by the imbalance between antioxidants and pro-oxidants. Several studies have suggested that this redox imbalance is involved in the pathogenesis of blood pressure elevation [9,10,11] as a part of the occurrence of endothelial dysfunction [12].

The role of ROS can be involved in several contributory mechanisms leading to the development of hypertension, such as increased intracellular calcium concentration or decreased NO bioavailability. Recent advances in the redox-sensitive cell signaling metabolic pathways may provide new therapeutic targets in the treatment of this pathology. 

In turn, basic and clinical studies have demonstrated the association of oxidative stress and essential hypertension. Thus, subjects with hypertension produce excessive amounts of ROS and show abnormal antioxidant profiles [13]. It is worth noting that these associations are expressed in the values of biomarkers of oxidative stress as both systolic and diastolic blood pressures of hypertensive and normotensive are positively correlated with plasma F2-isoprostane levels and negatively correlated with plasma total antioxidant capacity [14]. In addition, cultured vascular smooth muscle cells and isolated arteries from hypertensive rats and humans show enhanced ROS production, amplified redox-dependent signaling, and reduced antioxidant bioactivity. Therefore, it is plausible to consider the role of antioxidants within the available therapeutic resources for hypertensive patients. Moreover, it is of interest that the effects of classical antihypertensive drugs, such as β-adrenergic blockers, ACE inhibitors, angiotensin receptor blockers, and calcium channel blockers, may be partly mediated by decreasing vascular oxidative stress.

### 3.1. Experimental Approach between Hypertension and Oxidative Stress

Several experimental models have contributed to establishing a relationship between ROS and blood pressure elevation, such as those involving the role of NADPH oxidase in the development of salt-sensitive hypertension [15]. Thus, it was found that the expression of the subunit p67(phox) of this enzyme was increased in response to a high-salt diet in the outer medulla of Dahl rats. At the genetic level, mutations in the promoter region of the SS allele p67 account for higher promoter activity. Interestingly, angiotensin II type 1 receptor antagonists protected against vascular damage to subjects having genetically enhanced sodium-sensitive blood pressure [16]. The vascular impairment has been related to the increased production of superoxide anion radical since this ROS reacts extremely rapidly with NO to produce peroxynitrite, thereby inducing vasoconstriction by elevating vascular resistance [17].

Recent studies indicate that increased oxidative stress at the level of the vascular wall is the major mediator of endothelial injury in the pathology of hypertension. Accordingly, increased production of pro-oxidants such as superoxide anion and hydrogen peroxide, reduced nitric oxide synthesis, and decreased bioavailability of antioxidants are associated to inflammation, hypertrophy, proliferation, apoptosis, fibrosis, and angiogenesis, among other vascular wall impairments, all of which are important processes contributing to endothelial dysfunction and cardiovascular remodeling in hypertension.

### 3.2. Antihypertensive Effects of Antioxidants

Together with taking into account the role of ROS in the pathophysiology of hypertension, the view of the potential effects of antioxidants as pharmacological agents leading to counteract blood pressure elevation has been suggested from various sources [18]. Thus, antioxidant-rich diets have resulted in lowering blood pressure in hypertensive subjects, and supplements of antioxidant vitamins reduced both systolic and diastolic blood pressure in patients with essential hypertension [19]. Naturally occurring antioxidants, such as wine polyphenols have also been considered as positive modulators of endogenous antioxidant defense systems [20]. Several preclinical studies and clinical trials have indicated that antioxidant therapy is important for the management of hypertension, using antioxidant compounds such as ascorbic acid (vitamin C), alpha-tocopherol (vitamin E) and polyphenols, and some antihypertensive drugs are now in clinical use (*e.g.*, ACEIs, ARBs, novel B-blockers, dihydropyridine CCBs), which have anti-oxidative pleiotropic effects. The antihypertensive effects of some of the most important antioxidants will be provided below.

#### 3.2.1. Vitamin C

Most of the studies in hypertensive patients have communicated an inverse relationship between plasma vitamin C levels and values of arterial pressure. This is consistent with experimental data showing that ascorbate exerts up-regulation of eNOS and down-regulation of NADPH oxidase, two enzymes closely related to the modulation of vasomotor tone [21]. In a randomized, double-blind, placebo-controlled study, it was demonstrated that treatment of hypertensive patients with vitamin C lowered blood pressure. More recently, it was found that vitamin C supplementation resulted in a significant vasodilatory effect in patients with essential hypertension [22]. The reduction of blood pressure could be explained based on the ability of vitamin C to augment the formation of PGE1, PGI2, NO, lipoxin A4, and restoring arachidonic acid content to normal. These effects are in agreement with the cytoprotective, vasodilator, and anti-aggregator actions of vitamin C in type 2 diabetes mellitus and hypertension [23]. It has been suggested that ascorbate increases intracellular concentrations of BH4, an eNOS co-factor that promotes the production of nitric oxide [24]. However, the results of clinical trials are inconsistent, probably because their methods are heterogeneous. Thus, more studies are still lacking to support robust data making suitable the clinical use of vitamin C as an antihypertensive agent.

#### 3.2.2. Vitamin E

Alpha-tocopherol is a lipid-soluble molecule able to modulate mitochondrial generation of free radicals in a dose-dependent manner to exert its antioxidant effect. In addition, from epidemiological data, it has been reported that high vitamin E intake associates with a lower incidence of cardiovascular diseases [25]. Nevertheless, other studies could not demonstrate the beneficial effects of vitamin E in patients having cardiovascular diseases. Unexpectedly, a clinical trial demonstrated that vitamin E supplementation caused an increase in blood pressure and cardiac frequency in type 2 diabetic patients [26]. Likely, vitamin E itself is not capable of counteracting all components of oxidative stress acting in the development of hypertension. In accordance with this view, attempts to treat blood pressure elevation have been made through a trial with the associations of vitamins C and E administered to never-treated patients with essential hypertension [27]. Following the administration of vitamins C+E, patients with essential hypertension showed lower levels of both systolic and diastolic pressures, thus suggesting intervention with antioxidants as an adjunct therapy for hypertension. Although emerging evidence suggests that vitamin E may contribute to blood pressure improvement, data are still controversial, and more studies are needed to draw definitive conclusions.

#### 3.2.3. Polyphenols

Polyphenols might protect the cardiovascular system by improving endothelial function. The mechanism whereby these compounds could be able to counteract vasoconstriction include an increase in eNOS activity, prevention of cyclooxygenase-dependent formation of endothelium –derived contracting factors [28], ROS scavenging, inhibition of NADPH, and xanthine oxidases, and chelation of metal ions, processes altogether aimed to increase the NO bioavailability [29]. Activation of eNOS is mostly caused by an increase in the free cytosolic calcium concentration in endothelial cells. The activation of eNOS is mediated by the PI3-kinase/Akt pathway, responsible for the response to shear stress, a mechanism triggered by eNOS phosphorylation at SER 1177 position. Resveratrol, or 3,5,4′-trihydroxy-trans-stilbene, a phenolic substance present in grapes, wine, peanuts, and berries, suitable to attenuate oxidative stress and inflammation, has demonstrated numerous vasculoprotective effects. It was demonstrated that resveratrol moderately diminishes systolic blood pressure in hypertensive patients [30]. However, there are still many unknowns, such as safety, dosage, and mechanism.

#### 3.2.4. Antioxidant Effects of Antihypertensive Therapy

Although the mechanisms underlying the pathological process of blood pressure elevation are not well elucidated, cumulated data point out that oxidative stress plays a key role in the pathophysiology of hypertension [31]. In addition, the pharmacological effect of antihypertensive drugs is linked to an interference of the cell signaling process leading to a prooxidant condition. However, up to date, data do not allow to recommend the antihypertensive use of a single antioxidant as a monotherapy. Nevertheless, data seems to suggest that the adjunct administration of antioxidant supplements could lead to a reinforcement of the therapeutic effects of the current antihypertensive drugs. This resource could be useful to be applied in resistant hypertension, a condition needing the optimally tolerated doses of 3 or even more different classes of pharmacological agents, including a diuretic. Interestingly, most antihypertensive drugs are able to exert antioxidant effects (Table 1). Therefore, it seems reasonable to expect an additive or synergistic effect between antioxidants and antihypertensive drugs, a paradigm warranting further exploration.

### 3.3. Combined Antioxidants Strategy

Although an association of different antioxidants providing individual mechanisms should be expected, and therein possible enhanced cardioprotection, this principle has not been appropriately applied in clinical studies. In addition, the drug resistance to antihypertensive effects has been extensively counteracted through the association of 2 or more drugs, and no attempts to include antioxidants in these associations have been made, even though it is well known that most of the currently used antihypertensive drugs have antioxidant properties. Therefore, the association of drugs with naturally occurring innocuous antioxidants, such as vitamins C and E or some polyphenols, needs to be tested to achieve additive or synergistic cardioprotection and optimize the antihypertensive effects of the pharmacological therapy. Moreover, the association of vitamin C (1 g/day) plus vitamin E (400 IU/day) resulted in significant blood pressure lowering in never-treated men with essential hypertension [27], thus supporting this proposal and suggesting the antioxidant combination as an adjunct antihypertensive therapy. Nevertheless, more clinical trials are still lacking in drawing definitive conclusions.

## 4. Acute Myocardial Infarction

Acute myocardial infarction (AMI) is the leading cause of death worldwide. AMI complications have decreased with the development of percutaneous coronary angioplasty (PCA) treatment, which effectively restores the blood flow to the area previously subjected to ischemia [48]. Paradoxically, the restoration of blood flow to the ischemic zone leads to a massive ROS production, which generates rapid and severe damage to biomolecules, in a phenomenon called myocardial reperfusion injury (MRI) [49]. MRI is associated with multiple complications such as lethal reperfusion, no-reflow phenomenon, myocardial stunning, and reperfusion arrhythmias. Despite significant advances achieved in the understanding of the mechanisms accounting for MRI, it remains an unsolved problem.

ROS not only exert their actions by directly modifying organic molecules but are also involved in the regulation of the expression of several genes [50]. NF-ĸB and AP-1, both of which can experience ROS-mediated activation, stimulate the transcription of pro-inflammatory cytokines that activate several cell death pathways [51]. Oxidative stress and inflammation have important molecular bridges that are activated in the presence of ROS, leading to the activation of mechanisms that cause MRI and heart tissue remodeling. Among those molecules, the most studied has been the transcriptional factor NF-ĸB, a factor that responds to changes of the cellular oxidative state, ischemia-reperfusion, and inflammatory molecules [52]. When NF-ĸB is activated, for example, in the presence of ROS by phosphorylation of its inhibitory cofactor (Iĸ-B), it bonds to a DNA response element and promotes the transcription of genes involved in inflammatory and pro-fibrotic response, such as IL-6, which transforms growth factors TGF-β and TNF-α [53]. 

Despite a molecular basis and in vitro evidence supporting the use of antioxidants to prevent MRI, clinical evidence continues to be controversial. Many antioxidants have been used for the prevention of MRI, and those with the most evidence are ascorbate, N-acetylcysteine, and deferoxamine.

### 4.1. Ascorbate

Ascorbate is an essential antioxidant that performs its roles in different cell locations by acting in water-soluble components [54]. The most studied mechanism in which vitamin C acts is partly based on its ability to directly reduce ROS [55]. Besides its ROS scavenging actions, ascorbate exerts a complex modulation of numerous enzymes involved in ROS production, endothelial dysfunction, platelet aggregation, and smooth muscle cell tone regulation [56]. The four most important mechanisms whereby ascorbate modulates the endothelial function are NADPH down-regulation and the up-regulation of eNOS, phospholipase A2, and antioxidant enzymes. NADPH oxidase can be directly down-regulated by ascorbate [57]. Ascorbate could be involved in the transcriptional and post-transcriptional modulation of NADPH oxidase [58] as well as in its synthesis [59]. In the presence of oxidative stress, eNOS is mostly in its uncoupled form, which leads to endothelial dysfunction. In this context, ascorbate has been shown to increase eNOS activity by preventing the oxidation of BH4 and by inhibiting the p47phox subunit expression [59]. Therefore, ascorbate increases NO synthesis, reduces ROS formation, and contributes to vascular tone regulation [56]. Some studies have demonstrated a positive correlation between antioxidant vitamin and antioxidant enzyme activity, particularly SOD. The mechanisms underlying these findings are not well explained, but it is plausible to hypothesize the existence of transcriptional and post-transcriptional events involved in the up-regulation of those antioxidant enzymes [55]. 

Ascorbate counteracts and prevents the oxidation of lipids, proteins, and DNA, subsequently protecting their structure and biological function. Together with glutathione, ascorbate constitutes a primary line of defense against ROS [58]. Ascorbate can recycle α-tocopherol in membranes by reducing the α-tocopheroxyl radical back to α-tocopherol.

Our group developed a randomized clinical trial (RCT) in patients with AMI undergoing PCA, where massive doses of ascorbate (or placebo) were administered prior to PCA. Patients treated with ascorbate prior to myocardial reperfusion showed a better recovery of ejection fraction at 2–3 months (measured by cardiac magnetic resonance) and significantly higher myocardial perfusion after PCA (TIMI-myocardial perfusion grade) than placebo patients, with no differences in infarct size [60].

### 4.2. N-Acetylcysteine (NAC)

Despite numerous studies and clinical trials, the effects of NAC are clouded in controversy, and its pharmacological mechanism has not yet been fully clarified. However, there is plenty of evidence regarding its mechanism of action. First of all, NAC’s main feature is its capacity to act as a precursor for the synthesis of reduced glutathione (GSH) synthesis, thus replenishing GSH that has become depleted through the use of this peptide in detoxification routes [61]. NAC acts indirectly through the chelation of metal ions such as catalytic iron [62], suppressing its capability of mediating Fenton’s reaction, thus ameliorating the possibility of the formation of hydroxyl radicals. Current evidence agrees on the capability of NAC to act as an inhibitor of NF-κB [63].

NAC has been widely used in different experimental and clinical settings to counteract oxidative stress. It has been demonstrated that NAC in combination with nitroglycerin and streptokinase is associated with significantly less oxidative stress and improved preservation of left ventricular function [64]. However, it has also been reported that a high-dose of NAC prior to PCA, although it reduces oxidative stress, does not provide an additional advantage in the prevention of myocardial reperfusion injury [65]. Additionally, an interesting study showed that administration of NAC in combination with streptokinase significantly diminishes oxidative stress and improves left ventricular function in patients with acute myocardial infarction [66]. A study using a rat model of myocardial ischemia-reperfusion injury demonstrates that treatment with continuous infusion of NAC before and after coronary occlusion produces a significant limitation of the infarct and allows the recovery of the decreased total glutathione [67]. The NACIAM trial [68] demonstrated a protective effect with the use of high doses of NAC in combination with a nitric oxide donor in patients with AMI. Ascorbate consumes GSH to exert its antioxidant activity. High doses of ascorbate might be associated with a decrease in cellular GSH reserves [69]. For this reason, NAC—a known GSH-donor—may also have synergistic effects with high doses of ascorbate [70].

### 4.3. Deferoxamine (DFO)

Given the known role of iron in lethal reperfusion, iron chelators have been tested to ameliorate this injury. One of the most frequently used drugs for this purpose is DFO. Some performed studies reported a decrease in the infarct size when they used DFO during the reperfusion, suggesting that iron-catalyzed production of ROS contributes to cardiomyocyte necrosis in MRI. Studies have described the improved recovery of myocardial function after ischemia, by using iron chelation. Paraskevaidis et al. suggested that DFO infusion was able to reduce myocardial stunning after elective coronary artery bypass grafting and to improve long-term ejection fraction [71]. Chan et al. studied patients with STEMI to intravenous DFO during PCA. The serum iron levels and lipid peroxidation biomarkers were reduced in the DFO-group without differences in the infarct size [72]. These results are in agreement with the involvement of ferroptosis, an iron-dependent form of non-apoptotic cell death [73,74] in the cardiac injury occurring during reperfusion, as demonstrated in a rat model [75], a relevant finding for the preventive therapy in AMI patients subjected to PCA.

### 4.4. Other Antioxidants

Allopurinol, a xanthine oxidase inhibitor, can decrease infarct size and improve the recovery of left ventricular function in animal models of MRI [76]. Melatonin is an endogenously produced molecule that protects against oxidative stress. Melatonin efficiently detoxifies ROS and RNS, and it also up-regulates antioxidant enzymes and down-regulates pro-oxidant enzymes [77]. The mechanism of protection of melatonin appears to involve the inhibition of mitochondrial permeability transition pore opening via prevention of cardiolipin peroxidation [78]. Melatonin reduces the infarct size area and the incidence of reperfusion arrhythmias [79] in acute myocardial infarction. Adenosine triphosphate sensitive potassium channel openers (KATP channel) exert cardioprotective effects in AMI, and these effects are mediated through mimicking of ischemic preconditioning [80]. Nicorandil is a nicotinamide ester that possesses K- ATP channel-activating and nitrate-like properties. Several studies have demonstrated that nicorandil could reduce infarct size and improve functional and clinical outcomes after AMI when administered adjunctively to PCA [81].

### 4.5. Combined Antioxidants Strategy

There is major evidence with respect to the contribution of oxidative stress to reperfusion injury in AMI. Despite the many significant advances in the understanding of the mechanisms of MRI, it remains an unsolved problem. Although promising results have been obtained in experimental studies, these benefits have not been translated into clinical settings. It is possible that a combined approach, based on the pharmacokinetic properties of antioxidants could be successful in preventing MRI. We propose that the association of ascorbate, NAC, and DFO could be a promising combination of antioxidants to prevent MRI, which warrants evaluation in RCT.

## 5. Postoperative Atrial Fibrillation

Atrial fibrillation (AF) frequently occurs after cardiac surgery with extracorporeal circulation, ranging from approximately 20–30% for patients in the early postoperative period of a coronary artery bypass graft surgery [82], 30–45% after valve surgery [83], up to 45–65% for combined valve and coronary artery procedures [83]. Postoperative atrial fibrillation has major adverse consequences for patients and the healthcare system, with increased risk of perioperative complications such as stroke, kidney failure, infections, longer intensive care unit and total hospital stay, higher in-hospital mortality, as well impaired long-term prognosis with increased rates of death after hospital discharge [84,85,86]. The problem of POAF is extensively acknowledged, yet the underlying mechanisms that lead to the onset and persistence of AF have been difficult to elucidate, and highly effective interventions are still lacking. Considering the relative inefficacy and side effects of classic antiarrhythmic drugs, novel efforts focus on preventive strategies targeting the AF substrate.

Inflammation and oxidative stress have long been associated with AF in animal and clinical settings, and several studies have further supported this notion in recent years [87,88,89,90,91,92,93,94,95]. In on-pump cardiac surgery, particularly, a yet growing body of evidence underscores the major role of inflammation and oxidative stress [96,97,98,99] as likely key pathophysiologic pathways promoting atrial electrical remodeling, which leads to a shortened atrial refractory period and delayed atrial conduction [100], thus increasing susceptibility to atrial fibrillation [101,102]. Because in on-pump cardiac surgery ischemia-reperfusion inevitably occurs, the formation of ROS takes place accordingly, causing oxidative stress and systemic inflammatory response. ROS-induced lipid peroxidation, protein carbonylation and/or nitration, and DNA oxidation are well-established mediating mechanisms of oxidative damage in cardiac tissue. Of note, oxidative stress-induced proinflammatory pathways further amplify the deleterious consequences of ischemia-reperfusion. Myocardial oxidative mechanisms and high ROS-induced cell death ultimately lead to cardiomyocyte alterations, structural modifications in cardiac tissue, and atrial remodeling with aberrations in impulse generation, propagation, duration, and configuration of individual cardiac action potentials. 

Noteworthy, end of surgery and postoperative day 2 biomarkers demonstrated relatively linear associations with POAF in 551 cardiac surgery patients of the OPERA Trial [103]. These important data add to the expanding evidence that oxidative stress contributes to the mechanism of POAF development [104,105], accounting for the cellular basis of this cardiac rhythm disorder [106,107]. Thus, several studies [108,109,110,111,112,113] aimed to modulate oxidative stress by means of antioxidant-driven strategies, which have shown promising yet inconsistent results. Herein we discuss findings on the therapeutic potential of antioxidants-based pharmacological strategies aimed at reducing the incidence of POAF.

### 5.1. Omega-3 Polyunsaturated Fatty Acids (n-3 PUFA)

For the prevention of POAF, numerous attempts have been made to introduce the cardioprotective potential of n-3 PUFA against ischemia-reperfusion injury [114,115,116,117,118,119]. The supporting rationale is that, among other mechanisms [120], omega-3 PUFA are known to induce low to moderate increments in ROS levels, which leads to a heightened endogenous antioxidant capacity by up-regulating cardiac antioxidant enzymes, e.g., catalase and glutathione peroxidase, through activation of the nuclear factor erythroid 2–related factor 2 transcription factor. 

Previous observational and interventional studies [121] suggesting beneficial effects of marine-derived n-3 PUFA on the risk of cardiac arrhythmias supported the performance of several randomized controlled trials (RCT) to reduce the incidence of POAF over the last 15 years [122,123,124,125,126,127,128,129,130,131]. The results have been rather contradictory, with studies reporting a significant inverse association [122,123,124,130] or neutral findings [125,126,127,128,129,131]. Further analyses combining data from two of these interventional studies originally reporting neutral findings, have also suggested a U-shaped association, as observed between red blood cells levels of docosahexaenoic acid (DHA) and risk of POAF incidence [132]. Even a negative association came to the attention of researchers from an observational study performed around the time most of the interventional studies tended to suggest a lack of a beneficial effect [133,134], leaving unanswered the question of whether n-3 PUFA could prevent POAF. 

Nine meta-analyses have aimed to answer this question [135,136,137,138,139,140,141,142,143], in turn, providing contradictory conclusions. While the first meta-analyses tended to suggest a beneficial effect of n-3 PUFA supplementation on POAF incidence [137,140,141], after the OPERA Trial —which is the RCT with the largest study population—was published, the conclusion and overall impression temporarily tipped towards no impact of n-3 PUFA supplementation on POAF incidence [135,137,138,141,142,143]. It should be taken into account, however, that the OPERA Trial [129], largely driving this seemingly settled conclusion, as well as other RCT with negative findings [126,128], performed n-3 PUFA supplementation only postoperatively or insufficiently before cardiac surgery to result in adequate incorporation of n-3 PUFA in sarcolemmal myocardial membranes [144]. The lack of findings may also be explained by the EPA:DHA supplementation ratio. It should be realized that several studies have shown a greater anti-arrhythmic potential of DHA compared to EPA [145,146,147,148], which in the cardiac postoperative context is supported by the observation that the EPA:DHA ratio 1:2 was used in studies with positive findings [123] whereas higher ratios were used in studies with negative findings [126,127,129,130]. This notion has also been supported by observations derived from a subgroup analysis of meta-analyses published thereafter [141,143]. Furthermore, considering the proposed upper threshold for the anti-arrhythmic effect of n-3 PUFA [149], it may also be proposed that pre-treatment levels—not investigated in the relevant RCT—may have worked as effect-modifier of the intervention [150], limiting the effect size observed by supplementing study populations with relatively higher –closer to the threshold– n-3 PUFA levels at baseline. 

Most recently, the debate has been resumed due to the report of two meta-analysis, including 17 RCTs (n = 3611) [151] and 14 RCTs (n = 3570) [152], which found significant POAF reduction with PUFA vs controls [152]. 

### 5.2. Vitamin C

Vitamin C counterbalances oxidative stress due to its superoxide anion scavenging activity and its capacity to limit ROS production via down-regulation of NADPH oxidase, which is the major cardiovascular enzymatic source of ROS. Several studies have aimed to validate its ability to counterbalance oxidative damage and decrease POAF incidence, with positive [153,154,155,156,157] and neutral findings [158,159,160,161,162].

In a previous systematic review [139] and in 4 different meta-analyses, including 8 to 15 RCTs conducted between 2016 and 2017 [163,164,165,166], vitamin C was shown to significantly reduce the occurrence of POAF. Noteworthy, although Baker et al. concluded that perioperative administration of ascorbic acid to patients undergoing cardiac surgery was associated with a reduced frequency of POAF [166], it came to the attention of the research community [167] that two seemingly large RCTs [168,169] performed in the United States (USA) were not included in these analyses. It should be realized, however, that the results of those RCTs were actually not published because the corresponding research groups did not seek publication due to their negative findings [167]. In fact, the results of these unpublished RCTs were obtained and reported by Hemilä [167] upon personal request and communication with the corresponding researchers. Failing to account for the results of these studies, as well as the results of a negative RCT published solely in the form of an abstract [170], introducing publication bias, may have, in turn, biased the overall results of the aforementioned meta-analyses [167]. The influence of study location on the beneficial effect of vitamin C intervention strategies was thereafter clearly distinguished in the following meta-analyses by Hemilä et al. [171]. It was shown that vitamin C reduced the incidence of POAF outside the USA, however, this finding did not hold true for studies performed in the USA, underscoring a context-dependency of the proposed effect, which should be taken into account in future studies [171]. Next, the most recent and comprehensive meta-analyses studying the effect of vitamin C in cardiac surgery patients was recently published [172], including a total of 2008 patients in 19 RCT, herein accounting for the results of the RCTs performed in the USA, either published in the form of an abstract [170] or unpublished [168,169]. Hill et al. found that vitamin C lowered the incidence of atrial fibrillation, as well as other relevant parameters closely related to the patient’s recovery (e.g., ventilation time, length of stay in the intensive care unit, and total hospital stay), underscoring the pleiotropic potential of vitamin C [172], further supporting a preceding and similarly designed meta-analysis [173]. It should be noted that due to heterogeneity regarding types of open-heart surgery and vitamin C administration, an overall summary of average treatment effect across trials was provided by means of random-effects meta-analyses, yet the overall quality of available evidence remains limited by heterogeneity in outcome definition, measurement, and reporting [172]. A subgroup analysis according to the route of vitamin C administration found that the effect of the intervention was statistically significant in the group receiving intravenous vitamin C, while it was not in patients receiving oral vitamin C [172]. On the other hand, subgroup analyses to investigate any possible influence of the control group (placebo or standard of care) found no evidence of a treatment effect between subgroups, as the treatment effect was significant in both groups. 

Hence, the current body of evidence supports the recommendation of intravenous vitamin C interventional strategies for the prevention of POAF in on-pump cardiac surgery as an adjunct to the current standard of care.

### 5.3. Combined Antioxidants Strategy

Our group has previously shown that combination therapy of n-3 PUFA with vitamins C and E is effective in reducing oxidative stress and inflammation biomarkers in patients undergoing on-pump cardiac surgery and effective in the prevention of POAF [174,175,176]. In 2013 our group published the results of a study performed in 203 cardiac surgical patients randomized to a combined regimen of n-3 PUFA, vitamin C, and vitamin E [176]. A significantly reduced incidence of POAF was found in the group receiving the combined antioxidant strategy therapy compared to controls (9.7% vs. 32%, RR 0.28, 95% CI 0.14–0.56, *p* < 0.001). These findings were further supported by a meta-analysis of 11 RCTs with 3137 patients showing that the use of n-3 PUFA alone did not reduce the incidence of POAF compared with the control (OR: 0.76; 95% confidence interval [CI]: 0.57–1.03; *p* = 0.08; I(2) = 52%) [143]. The authors concluded that a combined antioxidants strategy with n-3 PUFA, vitamin C, and vitamin E was effective in the prevention of POAF while PUFA alone is not. 

We propose to test the hypothesis that the simultaneous use of these antioxidants should enhance the effectiveness of the treatment of the diseases hereby reviewed through the use of these safe, low-cost, well-characterized, and easily available substances. Accordingly, further studies are being performed by us to investigate this paradigm.

## 6. Discussion

It has been recognized that oxidative stress represents a unifying mechanism that plays a key role in the development of cardiovascular disease. Although ROS and RNS play a physiological modulation of the vascular wall homeostasis, increased ROS levels lead to a cascade of events involving both structural and functional impairments. Here, we reported cumulated evidence for the involvement of oxidative stress in the pathophysiology of hypertension, acute myocardial infarction, and postoperative atrial fibrillation. Ischemia followed by reperfusion can trigger even worst deleterious oxidative damage. Despite the plausibility of antioxidant supplementation as therapeutic agents aimed to attenuate this injury, the translation of experimental data to clinical studies has been disappointing. This lack of coherence is likely due to the characteristics of therapeutic strategies performed in most clinical trials, mainly based on the supplementation with monotherapies of individual antioxidants. Nevertheless, the complex multifactorial nature of cardiac damage caused by ischemia-reperfusion events needs a multitarget antioxidant therapy to achieve optimal cardioprotection caused by various mechanisms that all together might give rise to additive or synergic protective effects. Accordingly, the pharmacological management of resistant hypertension has been successful through the association of 2 or more drugs, sharing the antioxidant effect as a major characteristic associated with their antihypertensive action. In agreement, we have previously shown that a supplementation approach with the association of n-3 PUFA, vitamin C, and vitamin E increased antioxidant potential, attenuated oxidative stress and inflammation, and favorably affected POAF [176] (Table 2). Attempts to reach similar beneficial effects by means of a multitarget antioxidant strategy, which may give rise to additive or synergic protective effects to achieve optimal cardioprotection, need to be further explored, which provides a novel proposal for researchers, as an adjunct safe, easily available, and low-cost therapy to improve or enhance the pharmacological effect of the drugs, as well as the effects of the combination of antioxidants themselves. 

## Figures and Tables

**Table 1:** Antioxidant mechanisms are involved in the pharmacological effects of currently used antihypertensive drugs.

Group: Drugs	Major Antioxidant Mechanism	Reference
Angiotensin-converting enzyme inhibitors: Enalapril Benazepril	Free radical scavenging NADPH oxidase inhibition Decreased ROS generation Increased NO bioavailability Increased SOD activity	de Cavanagh et al., 2011 [32] Deoghare and Kantharia, 2013 [33] Chandran et al., 2014 [34] Yusoff et al., 2017 [35]
Angiotensin receptor Antagonists: Losartan Telmisaran	NADPH oxidase down-regulation Decreased ROS Increased NO bioavailability	Chopra et al., 1990 [36] Schiffrin et al., 2010 [37] Cameron et al., 2016 [38] Donnarumma et al., 2016 [39]
Novel beta Blockers: Carvedilol Nebivolol	Increased NO bioavailability NADPH oxidase inhibition Reduced lipid peroxidation Reduced leukocyte ROS production	Bowman et al., 1994 [40] Mollnau et al., 2003 [41] Münzel et al., 2009 [42] Zepeda et al., 2012 [43]
Dihydropyridine calcium channel blockers: Nifedipine Benidipine	Decreased ROS NADPH oxidase inhibition Reduced lipid peroxidation	Lupo et al., 1994 [44] Brovkovych et al., 2001 [45] Virdis et al., 2011 [46] Mason 2012 [47]

ACEIs, angiotensin I converting enzyme inhibitors; NADPH, reduced nicotinamide adenine dinucleotide phosphate; NO, nitric oxide; NOS, nitric oxide synthase; ROS, reactive oxygen species; SOD, superoxide dismutase.

**Table 2:** Studies using a combined antioxidants strategy.

Study	Design	Study Population	Study Size	Dosage	Duration
Rodrigo et al., 2008 [27]	Randomized, double-blind placebo controlled	Essential hypertension	110	vitamin C (1 g/day) vitamin E (400 IU/day)	8 weeks
Castillo et al., 2010 [174]	Randomized, double-blind placebo controlled	Open heart surgery	95	n-3 PUFA 2 g/d (EPA/DHA = 1:2) vitamin C (1 g/day) vitamin E (400 IU/day)	PUFA: 7 days before surgery until discharge Vitamin C and E: 2 days before surgery until discharge
Rodrigo et al., 2012 [175]	Randomized, double-blind placebo controlled	Open heart surgery	152	n-3 PUFA 2 g/d (EPA/DHA = 1:2) vitamin C (1 g/day) vitamin E (400 IU/day)	PUFA: 7 days before surgery until discharge Vitamin C and E: 2 days before surgery until discharge
Rodrigo et al., 2013 [176]	Randomized, double-blind placebo controlled	Open heart surgery	203	n-3 PUFA 2 g/d (EPA/DHA = 1:2) vitamin C (1 g/day) vitamin E (400 IU/day)	PUFA: 7 days before surgery until discharge Vitamin C and E: 2 days before surgery until discharge

n-3 PUFA, omega-3 polyunsaturated fatty acids; EPA, eicosapentaenoic acid; DHA, docosahexaenoic acid.

## Data Availability

Not applicable.

## References

[1] Forouzanfar M.H., Alexander L., Bachman V.F., Biryukov S., Brauer M., Casey D., Coates M.M., Delwiche K., Estep K., Frostad J.J. (2015). Global, regional, and national comparative risk assessment of 79 behavioural, environmental and occupational, and metabolic risks or clusters of risks in 188 countries, 1990–2013: A systematic analysis for the Global Burden of Disease Study 2013. Lancet.

[2] Sinha N., Dabla P. (2015). Oxidative Stress and Antioxidants in Hypertension—A Current Review. Curr. Hypertens. Rev..

[3] Davidson S.M., Ferdinandy P., Andreadou I., Bøtker H.E., Heusch G., Ibáñez B., Ovize M., Schulz R., Yellon D.M., Hausenloy D.J. (2019). Multitarget Strategies to Reduce Myocardial Ischemia/Reperfusion Injury: JACC Review Topic of the Week. J. Am. Coll. Cardiol..

[4] Rodrigo R., Rodrigo R. (2009). Oxidative Stress and Antioxidants: Their Role in Human Disease.

[5] Sies H. (2017). Hydrogen peroxide as a central redox signaling molecule in physiological oxidative stress: Oxidative eustress. Redox Biol..

[6] Jain A., Mehra N., Swarnakar N. (2015). Role of Antioxidants for the Treatment of Cardiovascular Diseases: Challenges and Opportunities. Curr. Pharm. Des..

[7] Wang Y., Chun O.K., Song W.O. (2013). Plasma and dietary antioxidant status as cardiovascular disease risk factors: A review of human studies. Nutrients.

[8] Raedschelders K., Ansley D.M., Chen D.D.Y. (2012). The cellular and molecular origin of reactive oxygen species generation during myocardial ischemia and reperfusion. Pharmacol. Ther..

[9] Montezano A.C., Touyz R.M. (2012). Oxidative stress, Noxs, and hypertension: Experimental evidence and clinical controversies. Ann. Med..

[10] Montezano A.C., Dulak-Lis M., Tsiropoulou S., Harvey A., Briones A.M., Touyz R.M. (2015). Oxidative stress and human hypertension: Vascular mechanisms, biomarkers, and novel therapies. Can. J. Cardiol..

[11] Virdis A., Bacca A., Colucci R., Duranti E., Fornai M., Materazzi G., Ippolito C., Bernardini N., Blandizzi C., Bernini G. (2013). Endothelial dysfunction in small arteries of essential hypertensive patients: Role of cyclooxygenase-2 in oxidative stress generation. Hypertension.

[12] Vanhoutte P.M., Shimokawa H., Tang E.H.C., Feletou M. (2009). Endothelial dysfunction and vascular disease. Acta Physiol..

[13] Briones A.M., Touyz R.M. (2010). Oxidative stress and hypertension: Current concepts. Curr. Hypertens. Rep..

[14] Rodrigo R., Prat H., Passalacqua W., Araya J., Guichard C., Bächler J.P. (2007). Relationship between oxidative stress and essential hypertension. Hypertens. Res..

[15] Feng D., Yang C., Geurts A.M., Kurth T., Liang M., Lazar J., Mattson D.L., O’Connor P.M., Cowley A.W. (2012). Increased expression of NAD(P)H oxidase subunit p67 phox in the renal medulla contributes to excess oxidative stress and salt-sensitive hypertension. Cell Metab..

[16] Kosaka S., Pelisch N., Rahman M., Nakano D., Hitomi H., Kobori H., Fukuoka N., Kobara H., Mori H., Masaki T. (2013). Effects of angiotensin II AT1-receptor blockade on high fat diet-induced vascular oxidative stress and endothelial dysfunction in dahl salt-sensitive rats. J. Pharmacol. Sci..

[17] Zicha J., Dobešová Z., Kuneš J. (2001). Relative deficiency of nitric oxide-dependent vasodilation in salt-hypertensive Dahl rats: The possible role of superoxide anions. J. Hypertens..

[18] Rodrigo R., Gil D., Miranda-Merchak A., Kalantzidis G. (2012). Antihypertensive Role of Polyphenols. Advances in Clinical Chemistry.

[19] Rodrigo R., Bächler J.P., Araya J., Prat H., Passalacqua W. (2007). Relationship between (Na + K)-ATPase activity, lipid peroxidation and fatty acid profile in erythrocytes of hypertensive and normotensive subjects. Mol. Cell. Biochem..

[20] Rodrigo R., Miranda A., Vergara L. (2011). Modulation of endogenous antioxidant system by wine polyphenols in human disease. Clin. Chim. Acta.

[21] Ülker S., McKeown P.P., Bayraktutan U. (2003). Vitamins reverse endothelial dysfunction through regulation of eNOS and NAD(P)H oxidase activities. Hypertension.

[22] Guan Y., Dai P., Wang H., Wane D. (2020). Effects of Vitamin C supplementation on essential hypertension: A systematic review and meta-analysis. Medicine.

[23] Das U.N. (2019). Vitamin C for Type 2 Diabetes Mellitus and Hypertension. Arch. Med. Res..

[24] Al-Khudairy L., Flowers N., Wheelhouse R., Ghannam O., Hartley L., Stranges S., Rees K., Rees K. (2017). Vitamin C supplementation for the primary prevention of cardiovascular disease. Cochrane Database of Systematic Reviews.

[25] Kuwabara A., Nakade M., Tamai H., Tsuboyama-Kasaoka N., Tanaka K. (2014). The association between vitamin E intake and hypertension: Results from the re-analysis of the national health and nutrition survey. J. Nutr. Sci. Vitaminol..

[26] Ward N.C., Wu J.H.Y., Clarke M.W., Puddey I.B., Burke V., Croft K.D., Hodgson J.M. (2007). The effect of vitamin E on blood pressure in individuals with type 2 diabetes: A randomized, double-blind, placebo-controlled trial. J. Hypertens..

[27] Rodrigo R., Prat H., Passalacqua W., Araya J., Bächler J.P. (2008). Decrease in oxidative stress through supplementation of vitamins C and E is associated with a reduction in blood pressure in patients with essential hypertension. Clin. Sci..

[28] Kane M.O., Etienne-Selloum N., Madeira S.V.F., Sarr M., Walter A., Dal-Ros S., Schott C., Chataigneau T., Schini-Kerth V.B. (2010). Endothelium-derived contracting factors mediate the Ang II-induced endothelial dysfunction in the rat aorta: Preventive effect of red wine polyphenols. Pflugers Arch. Eur. J. Physiol..

[29] Nijveldt R.J., Van Nood E., Van Hoorn D.E.C., Boelens P.G., Van Norren K., Van Leeuwen P.A.M. (2001). Flavonoids: A review of probable mechanisms of action and potential applications. Am. J. Clin. Nutr..

[30] Breuss J.M., Atanasov A.G., Uhrin P. (2019). Resveratrol and its effects on the vascular system. Int. J. Mol. Sci..

[31] Ahmad K.A., Yuan Yuan D., Nawaz W., Ze H., Zhuo C.X., Talal B., Taleb A., Mais E., Qilong D. (2017). Antioxidant therapy for management of oxidative stress induced hypertension. Free Radic. Res..

[32] De Cavanagh E.M.V., Inserra F., Toblli J., Stella I., Fraga C.G., Ferder L. (2001). Enalapril attenuates oxidative stress in diabetic rats. Hypertension.

[33] Deoghare S., Kantharia N. (2013). Effect of atenolol and enalapril treatment on oxidative stress parameters in patients with essential hypertension. Int. J. Basic Clin. Pharmacol..

[34] Chandran G., Sirajudeen K.N.S., Nik Yusoff N.S., Swamy M., Samarendra M.S. (2014). Effect of the antihypertensive drug enalapril on oxidative stress markers and antioxidant enzymes in kidney of spontaneously hypertensive rat. Oxid. Med. Cell. Longev..

[35] Yusoff N.S.N., Mustapha Z., Sharif S.E.T., Govindasamy C., Sirajudeen K.N.S. (2017). Effect of antihypertensive drug treatment on oxidative stress markers in heart of spontaneously hypertensive rat models. J. Environ. Pathol. Toxicol. Oncol..

[36] Chopra M., McMurray J., Stewart J., Dargie H.J., Smith W.E. (1990). Free radical scavenging: A potentially beneficial action of thiol-containing angiotensin converting enzyme inhibitors. Biochem. Soc. Trans..

[37] Schiffrin E.L. (2010). Circulatory therapeutics: Use of antihypertensive agents and their effects on the vasculature. J. Cell. Mol. Med..

[38] Cameron A.C., Lang N.N., Touyz R.M. (2016). Drug Treatment of Hypertension: Focus on Vascular Health. Drugs.

[39] Donnarumma E., Ali M.J., Rushing A.M., Scarborough A.L., Bradley J.M., Organ C.L., Islam K.N., Polhemus D.J., Evangelista S., Cirino G. (2016). Zofenopril protects against myocardial ischemia-reperfusion injury by increasing nitric oxide and hydrogen sulfide bioavailability. J. Am. Heart Assoc..

[40] Bowman A., Chen C., Ford G. (1994). Nitric oxide mediated venodilator effects of nebivolol. Br. J. Clin. Pharmacol..

[41] Mollnau H., Schulz E., Daiber A., Baldus S., Oelze M., August M., Wendt M., Walter U., Geiger C., Agrawal R. (2003). Nebivolol prevents vascular NOS III uncoupling in experimental hyperlipidemia and inhibits NADPH oxidase activity in inflammatory cells. Arterioscler. Thromb. Vasc. Biol..

[42] Münzel T., Gori T. (2009). Nebivolol: The somewhat-different beta-adrenergic receptor blocker. J. Am. Coll. Cardiol..

[43] Zepeda R.J., Castillo R., Rodrigo R., Prieto J.C., Aramburu I., Brugere S., Galdames K., Noriega V., Miranda H.F. (2012). Effect of Carvedilol and Nebivolol on Oxidative Stress-related Parameters and Endothelial Function in Patients with Essential Hypertension. Basic Clin. Pharmacol. Toxicol..

[44] Lupo E., Locher R., Weisser B., Vetter W. (1994). In vitro antioxidant activity of calcium antagonists against LDL oxidation compared with α-tocopherol. Biochem. Biophys. Res. Commun..

[45] Brovkovych V., Kalinowski L., Muller-Peddinghaus R., Malinski T. (2001). Synergistic antihypertensive effects of nifedipine on endothelium: Concurrent release of NO and scavenging of superoxide. Hypertension.

[46] Virdis A., Ghiadoni L., Taddei S. (2011). Effects of antihypertensive treatment on endothelial function. Curr. Hypertens. Rep..

[47] Mason R.P. (2012). Pleiotropic effects of calcium channel blockers. Curr. Hypertens. Rep..

[48] Magro M., Nauta S., Simsek C., Onuma Y., Garg S., Van Der Heide E., Van Der Giessen W.J., Boersma E., Van Domburg R.T., Van Geuns R.J. (2011). Value of the SYNTAX score in patients treated by primary percutaneous coronary intervention for acute ST-elevation myocardial infarction: The MI SYNTAXscore study. Am. Heart J..

[49] Yellon D.M., Hausenloy D.J. (2007). Myocardial Reperfusion Injury. N. Engl. J. Med..

[50] Kim Y.H., Lee J.H., Lim D.S., Shim W.J., Ro Y.M., Park G.H., Becker K.G., Cho-Chung Y.S., Kim M.K. (2003). Gene expression profiling of oxidative stress on atrial fibrillation in humans. Exp. Mol. Med..

[51] Bowie A., O’Neill L.A.J. (2000). Oxidative stress and nuclear factor-κB activation: A reassessment of the evidence in the light of recent discoveries. Proceedings of the Biochemical Pharmacology.

[52] Chandra J., Samali A., Orrenius S. (2000). Triggering and modulation of apoptosis by oxidative stress. Free Radic. Biol. Med..

[53] Liakopoulos O.J., Schmitto J.D., Kazmaier S., Bräuer A., Quintel M., Schoendube F.A., Dörge H. (2007). Cardiopulmonary and Systemic Effects of Methylprednisolone in Patients Undergoing Cardiac Surgery. Ann. Thorac. Surg..

[54] Levine M., Rumsey S.C., Daruwala R., Park J.B., Wang Y. (1999). Criteria and recommendations for vitamin C intake. J. Am. Med. Assoc..

[55] Guney M., Oral B., Demirin H., Karahan N., Mungan T., Delibas N. (2007). Protective effects of vitamins C and E against endometrial damage and oxidative stress in fluoride intoxication. Clin. Exp. Pharmacol. Physiol..

[56] Wu F., Tyml K., Wilson J.X. (2008). iNOS expression requires NADPH oxidase-dependent redox signaling in microvascular endothelial cells. J. Cell. Physiol..

[57] Newaz M.A., Yousefipour Z., Nawal N.N.A. (2005). Modulation of nitric oxide synthase activity in brain, liver, and blood vessels of spontaneously hypertensive rats by ascorbic acid: Protection from free radical injury. Clin. Exp. Hypertens..

[58] Gao F., Yao C.L., Gao E., Mo Q.Z., Yan W.L., McLaughlin R., Lopez B.L., Christopher T.A., Ma X.L. (2002). Enhancement of glutathione cardioprotection by ascorbic acid in myocardial reperfusion injury. J. Pharmacol. Exp. Ther..

[59] Rahmouni K., Correia M.L.G., Haynes W.G., Mark A.L. (2005). Obesity-associated hypertension: New insights into mechanisms. Hypertension.

[60] Ramos C., Brito R., González-Montero J., Valls N., Gormaz J.G., Prieto J.C., Aguayo R., Puentes Á., Noriega V., Pereira G. (2017). Effects of a novel ascorbate-based protocol on infarct size and ventricle function in acute myocardial infarction patients undergoing percutaneous coronary angioplasty. Arch. Med. Sci..

[61] Lee K.A., Roth R.A., LaPres J.J. (2007). Hypoxia, drug therapy and toxicity. Pharmacol. Ther..

[62] Joshi D., Mittal D.K., Shrivastava S., Shukla S. (2010). Protective role of thiol chelators against dimethylmercury induced toxicity in male rats. Bull. Environ. Contam. Toxicol..

[63] Lu Y., Qin W., Shen T., Dou L., Man Y., Wang S., Xiao C., Li J. (2011). The antioxidant N-acetylcysteine promotes atherosclerotic plaque stabilization through suppression of rage, MMPs and NF-κB in ApoE-deficient Mice. J. Atheroscler. Thromb..

[64] Arstall M.A., Yang J., Stafford I., Betts W.H., Horowitz J.D. (1995). N-acetylcysteine in combination with nitroglycerin and streptokinase for the treatment of evolving acute myocardial infarction: Safety and biochemical effects. Circulation.

[65] Thiele H., Hildebrand L., Schirdewahn C., Eitel I., Adams V., Fuernau G., Erbs S., Linke A., Diederich K.W., Nowak M. (2010). Impact of High-Dose N-Acetylcysteine Versus Placebo on Contrast-Induced Nephropathy and Myocardial Reperfusion Injury in Unselected Patients With ST-Segment Elevation Myocardial Infarction Undergoing Primary Percutaneous Coronary Intervention. The LIPSIA-N-ACC (Prospective, Single-Blind, Placebo-Controlled, Randomized Leipzig Immediate PercutaneouS Coronary Intervention Acute Myocardial Infarction. J. Am. Coll. Cardiol..

[66] Yesilbursa D., Serdar A., Senturk T., Serdar Z., Saǧ S., Cordan J. (2006). Effect of N-acetylcysteine on oxidative stress and ventricular function in patients with myocardial infarction. Heart Vessels.

[67] Abe M., Takiguchi Y., Ichimaru S., Tsuchiya K., Wada K. (2008). Comparison of the protective effect of N-acetylcysteine by different treatments on rat myocardial ischemia-reperfusion injury. J. Pharmacol. Sci..

[68] Pasupathy S., Tavella R., Grover S., Raman B., Procter N.E.K., Du Y.T., Mahadavan G., Stafford I., Heresztyn T., Holmes A. (2017). Early use of N-acetylcysteine with nitrate therapy in patients undergoing primary percutaneous coronary intervention for ST-segment-elevation myocardial infarction reduces myocardial infarct size (the NACIAM trial [N-acetylcysteine in acute myocardial infarction]). Circulation.

[69] Rodrigo R., Libuy M., Feliú F., Hasson D. (2013). Molecular basis of cardioprotective effect of antioxidant vitamins in myocardial infarction. Biomed. Res. Int..

[70] González-Montero J., Brito R., Gajardo A.I., Rodrigo R. (2018). Myocardial reperfusion injury and oxidative stress: Therapeutic opportunities. World J. Cardiol..

[71] Paraskevaidis I.A., Iliodromitis E.K., Vlahakos D., Tsiapras D.P., Nikolaidis A., Marathias A., Michalis A., Kremastinos D.T. (2005). Deferoxamine infusion during coronary artery bypass grafting ameliorates lipid peroxidation and protects the myocardium against reperfusion injury: Immediate and long-term significance. Eur. Heart J..

[72] Søndergaard L., Steinbrüchel D.A., Ihlemann N., Nissen H., Kjeldsen B.J., Petursson P., Ngo A.T., Olsen N.T., Chang Y., Franzen O.W. (2016). Two-year outcomes in patients with severe aortic valve stenosis randomized to transcatheter versus surgical aortic valve replacement: The all-comers nordic aortic valve intervention randomized clinical trial. Circ. Cardiovasc. Interv..

[73] Tang D., Chen X., Kang R., Kroemer G. (2021). Ferroptosis: Molecular mechanisms and health implications. Cell. Res..

[74] Lillo-Moya J., Rojas-Solé C., Muñoz-Salamanca D., Panieri E., Saso L., Rodrigo R. (2021). Targeting Ferroptosis against Ischemia/Reperfusion Cardiac Injury. Antioxidants.

[75] Tang L.J., Luo X.J., Tu H., Chen H., Xiong X.M., Li N.S., Peng J. (2020). Ferroptosis occurs in phase of reperfusion but not ischemia in rat heart following ischemia or ischemia/reperfusion. Naunyn Schmiedebergs Arch. Pharmacol..

[76] Kinugasa Y., Ogino K., Furuse Y., Shiomi T., Tsutsui H., Yamamoto T., Igawa O., Hisatome I., Shigemasa C. (2003). Allopurinol improves cardiac dysfunction after ischemia-reperfusion via reduction of oxidative stress in isolated perfused rat hearts. Circ. J..

[77] Dominguez-Rodriguez A., Abreu-Gonzalez P., Sanchez-Sanchez J.J., Kaski J.C., Reiter R.J. (2010). Melatonin and circadian biology in human cardiovascular disease. J. Pineal Res..

[78] Petrosillo G., Colantuono G., Moro N., Ruggiero F.M., Tiravanti E., Di Venosa N., Fiore T., Paradies G. (2009). Melatonin protects against heart ischemia-reperfusion injury by inhibiting mitochondrial permeability transition pore opening. Am. J. Physiol. Heart Circ. Physiol..

[79] Dominguez-Rodriguez A., Abreu-Gonzalez P., Garcia-Gonzalez M.J., Samimi-Fard S., Reiter R.J., Kaski J.C. (2008). Association of ischemia-modified albumin and melatonin in patients with ST-elevation myocardial infarction. Atherosclerosis.

[80] Han C.L., Sung G.A., Choi J.H., Tae K.L., Kim J., June H.K., Kook J.C., Taek J.H., Yung W.S., Lee S.K. (2008). Effect of intra-coronary nicorandil administration prior to reperfusion in acute ST segment elevation myocardial infarction. Circ. J..

[81] Fukuzawa S., Ozawa S., Inagaki M., Shimada K., Sugioka J., Tateno K., Ueda M. (2000). Nicorandil affords cardioprotection in patients with acute myocardial infarction treated with primary percutaneous transluminal coronary angiosplasty: Assessment with thallium-201/iodine-123 BMIPP dual SPECT. J. Nucl. Cardiol..

[82] Jawitz O.K., Gulack B.C., Brennan J.M., Thibault D.P., Wang A., O’Brien S.M., Schroder J.N., Gaca J.G., Smith P.K. (2020). Association of postoperative complications and outcomes following coronary artery bypass grafting. Am. Heart J..

[83] Butt J.H., Olesen J.B., Gundlund A., Kümler T., Olsen P.S., Havers-Borgersen E., Aagaard D.T., Gislason G.H., Torp-Pedersen C., Køber L. (2019). Long-term Thromboembolic Risk in Patients with Postoperative Atrial Fibrillation after Left-Sided Heart Valve Surgery. JAMA Cardiol..

[84] Phan K., Ha H.S.K., Phan S., Medi C., Thomas S.P., Yan T.D. (2015). New-onset atrial fibrillation following coronary bypass surgery predicts long-term mortality: A systematic review and meta-analysis. Eur. J. Cardio Thorac. Surg..

[85] Megens M.R., Churilov L., Thijs V. (2017). New-Onset Atrial Fibrillation After Coronary Artery Bypass Graft and Long-Term Risk of Stroke: A Meta-Analysis. J. Am. Heart Assoc..

[86] Kerwin M., Saado J., Pan J., Ailawadi G., Mazimba S., Salerno M., Mehta N. (2020). New-onset atrial fibrillation and outcomes following isolated coronary artery bypass surgery: A systematic review and meta-analysis. Clin. Cardiol..

[87] Monrad M., Sajadieh A., Christensen J.S., Ketzel M., Raaschou-Nielsen O., Tjønneland A., Overvad K., Loft S., Sørensen M. (2017). Long-term exposure to traffic-related air pollution and risk of incident Atrial Fibrillation: A cohort study. Environ. Health Perspect..

[88] Li J.Y., He Y., Ke H.H., Jin Y., Jiang Z.Y., Zhong G.Q. (2017). Plasma oxidative stress and inflammatory biomarkers are associated with the sizes of the left atrium and pulmonary vein in atrial fibrillation patients. Clin. Cardiol..

[89] Samman Tahhan A., Sandesara P.B., Hayek S.S., Alkhoder A., Chivukula K., Hammadah M., Mohamed-Kelli H., O’Neal W.T., Topel M., Ghasemzadeh N. (2017). Association between oxidative stress and atrial fibrillation. Heart Rhythm.

[90] Karam B.S., Chavez-Moreno A., Koh W., Akar J.G., Akar F.G. (2017). Oxidative stress and inflammation as central mediators of atrial fibrillation in obesity and diabetes. Cardiovasc. Diabetol..

[91] Scott L., Li N., Dobrev D. (2019). Role of inflammatory signaling in atrial fibrillation. Int. J. Cardiol..

[92] Nattel S., Heijman J., Zhou L., Dobrev D. (2020). Molecular Basis of Atrial Fibrillation Pathophysiology and Therapy: A Translational Perspective. Circ. Res..

[93] Yang X., An N., Zhong C., Guan M., Jiang Y., Li X., Zhang H., Wang L., Ruan Y., Gao Y. (2020). Enhanced cardiomyocyte reactive oxygen species signaling promotes ibrutinib-induced atrial fibrillation. Redox Biol..

[94] Sanchez A.M., Germany R., Lozier M.R., Schweitzer M.D., Kosseifi S., Anand R. (2020). Central sleep apnea and atrial fibrillation: A review on pathophysiological mechanisms and therapeutic implications. IJC Heart Vasc..

[95] Zhou X., Dudley S.C. (2020). Evidence for Inflammation as a Driver of Atrial Fibrillation. Front. Cardiovasc. Med..

[96] Zakkar M., Ascione R., James A.F., Angelini G.D., Suleiman M.S. (2015). Inflammation, oxidative stress and postoperative atrial fibrillation in cardiac surgery. Pharmacol. Ther..

[97] Youn J.Y., Zhang J., Zhang Y., Chen H., Liu D., Ping P., Weiss J.N., Cai H. (2013). Oxidative stress in atrial fibrillation: An emerging role of NADPH oxidase. J. Mol. Cell. Cardiol..

[98] Türker F.S., Doǧan A., Ozan G., Kjbar K., Erişir M. (2016). Change in free radical and antioxidant enzyme levels in the patients undergoing open heart surgery with cardiopulmonary bypass. Oxid. Med. Cell. Longev..

[99] Emelyanova L., Ashary Z., Cosic M., Negmadjanov U., Ross G., Rizvi F., Olet S., Kress D., Sra J., Jamil Tajik A. (2016). Selective downregulation of mitochondrial electron transport chain activity and increased oxidative stress in human atrial fibrillation. Am. J. Physiol. Heart Circ. Physiol..

[100] Omae T., Inada E. (2018). New-onset atrial fibrillation: An update. J. Anesth..

[101] Korantzopoulos P., Kolettis T.M., Galaris D., Goudevenos J.A. (2007). The role of oxidative stress in the pathogenesis and perpetuation of atrial fibrillation. Int. J. Cardiol..

[102] Bramer S., Van Straten A.H.M., Soliman Hamad M.A., Berreklouw E., Martens E.J., Maessen J.G. (2010). The impact of new-onset postoperative atrial fibrillation on mortality after coronary artery bypass grafting. Ann. Thorac. Surg..

[103] Wu J.H.Y., Marchioli R., Silletta M.G., Masson S., Sellke F.W., Libby P., Milne G.L., Brown N.J., Lombardi F., Damiano R.J. (2015). Oxidative Stress Biomarkers and Incidence of Postoperative Atrial Fibrillation in the Omega-3 Fatty Acids for Prevention of Postoperative Atrial Fibrillation (OPERA) Trial. J. Am. Heart Assoc..

[104] Anderson E.J., Efird J.T., Davies S.W., O’Neal W.T., Darden T.M., Thayne K.A., Katunga L.A., Kindell L.C., Ferguson T.B., Anderson C.A. (2014). Monoamine oxidase is a major determinant of redox balance in human atrial myocardium and is associated with postoperative atrial fibrillation. J. Am. Heart Assoc..

[105] Lubbers E.R., Murphy N.P., Mohler P.J. (2015). Defining the links between oxidative stress-based biomarkers and postoperative atrial fibrillation. J. Am. Heart Assoc..

[106] Xie W., Santulli G., Reiken S.R., Yuan Q., Osborne B.W., Chen B.X., Marks A.R. (2015). Mitochondrial oxidative stress promotes atrial fibrillation. Sci. Rep..

[107] Tse G., Yan B.P., Chan Y.W.F., Tian X.Y., Huang Y. (2016). Reactive oxygen species, endoplasmic reticulum stress and mitochondrial dysfunction: The link with cardiac arrhythmogenesis. Front. Physiol..

[108] Rodrigo R., Castillo R., Cereceda M., Asenjo R., Zamorano J., Araya J. (2007). Non-hypoxic preconditioning of myocardium against postoperative atrial fibrillation: Mechanism based on enhancement of the antioxidant defense system. Med. Hypotheses.

[109] Rodrigo R., Cereceda M., Castillo R., Asenjo R., Zamorano J., Araya J., Castillo-Koch R., Espinoza J., Larraín E. (2008). Prevention of atrial fibrillation following cardiac surgery: Basis for a novel therapeutic strategy based on non-hypoxic myocardial preconditioning. Pharmacol. Ther..

[110] Rodrigo R., Vinay J., Castillo R., Cereceda M., Asenjo R., Zamorano J., Araya J., Castillo-Koch R., Espinoza J., Larraín E. (2010). Use of vitamins C and E as a prophylactic therapy to prevent postoperative atrial fibrillation. Int. J. Cardiol..

[111] Liu T., Li G. (2010). Antioxidant interventions as novel preventive strategies for postoperative atrial fibrillation. Int. J. Cardiol..

[112] D’Oria R., Schipani R., Leonardini A., Natalicchio A., Perrini S., Cignarelli A., Laviola L., Giorgino F. (2020). The Role of Oxidative Stress in Cardiac Disease: From Physiological Response to Injury Factor. Oxid. Med. Cell. Longev..

[113] Rodrigo R. (2012). Prevention of postoperative atrial fibrillation: Novel and safe strategy based on the modulation of the antioxidant system. Front. Physiol..

[114] Savelieva I., Camm A.J. (2011). Polyunsaturated fatty acids for prevention of atrial fibrillation: A “fishy” story Secondary prevention studies. Europace.

[115] Ofman P., Peralta A., Hoffmeister P., Gaziano J.M., Djousse L. (2013). Do omega-3 fatty acids decrease the incidence of atrial fibrillation?. J. Atr. Fibrillation.

[116] Orso F., Fabbri G., Maggioni A. (2015). Pietro Upstream treatment of atrial fibrillation with n-3 polyunsaturated fatty acids: Myth or reality?. Arrhythmia Electrophysiol. Rev..

[117] Lomivorotov V.V., Efremov S.M., Pokushalov E.A., Karaskov A.M. (2016). New-Onset Atrial Fibrillation after Cardiac Surgery: Pathophysiology, Prophylaxis, and Treatment. J. Cardiothorac. Vasc. Anesth..

[118] Farías J.G., Carrasco-Pozo C., Carrasco Loza R., Sepúlveda N., Álvarez P., Quezada M., Quiñones J., Molina V., Castillo R.L. (2017). Polyunsaturated fatty acid induces cardioprotection against ischemia-reperfusion through the inhibition of NF-kappaB and induction of Nrf2. Exp. Biol. Med..

[119] Seo C., Michie C., Hibbert B., Davis D.R. (2020). Systematic review of pre-clinical therapies for post-operative atrial fibrillation. PLoS ONE.

[120] Rix T.A., Mortensen L.M., Schmidt E.B. (2012). Fish, Marine n−3 Fatty Acids, and Atrial Fibrillation—Experimental Data and Clinical Effects. Front. Physiol..

[121] Mozaffarian D., Psaty B.M., Rimm E.B., Lemaitre R.N., Burke G.L., Lyles M.F., Lefkowitz D., Siscovick D.S. (2004). Fish intake and risk of incident atrial fibrillation. Circulation.

[122] Heidt M.C., Vician M., Stracke S.K., Stadlbauer T., Grebe M.T., Boening A., Vogt P.R., Erdogan A. (2009). Beneficial effects of intravenously administered N-3 fatty acids for the prevention of atrial fibrillation after coronary artery bypass surgery: A prospective randomized study. Thorac. Cardiovasc. Surg..

[123] Calò L., Bianconi L., Colivicchi F., Lamberti F., Loricchio M.L., De Ruvo E., Meo A., Pandozi C., Staibano M., Santini M. (2005). N-3 fatty acids for the prevention of atrial fibrillation after coronary artery bypass surgery: A randomized, controlled trial. J. Am. Coll. Cardiol..

[124] Sorice M., Tritto F.P., Sordelli C., Gregorio R., Piazza L. (2011). N-3 polyunsaturated fatty acids reduces post-operative atrial fibrillation incidence in patients undergoing “on-pump” coronary artery bypass graft surgery. Monaldi Arch. Chest Dis. Card. Ser..

[125] Saravanan P., Bridgewater B., West A.L., O’Neill S.C., Calder P.C., Davidson N.C. (2010). Omega-3 fatty acid supplementation does not reduce risk of atrial fibrillation after coronary artery bypass surgery: A randomized, double-blind, placebo-controlled clinical trial. Circ. Arrhythmia Electrophysiol..

[126] Heidarsdottir R., Arnar D.O., Skuladottir G.V., Torfason B., Edvardsson V., Gottskalksson G., Palsson R., Indridason O.S. (2010). Does treatment with n-3 polyunsaturated fatty acids prevent atrial fibrillation after open heart surgery?. Europace.

[127] Farquharson A.L., Metcalf R.G., Sanders P., Stuklis R., Edwards J.R.M., Gibson R.A., Cleland L.G., Sullivan T.R., James M.J., Young G.D. (2011). Effect of dietary fish oil on atrial fibrillation after cardiac surgery. Am. J. Cardiol..

[128] Sandesara C.M., Chung M.K., Van Wagoner D.R., Barringer T.A., Allen K., Ismail H.M., Zimmerman B., Olshansky B. (2012). A Randomized, Placebo-Controlled Trial of Omega-3 Fatty Acids for Inhibition of Supraventricular Arrhythmias After Cardiac Surgery: The FISH Trial. J. Am. Heart Assoc..

[129] Mozaffarian D., Marchioli R., Macchia A., Silletta M.G., Ferrazzi P., Gardner T.J., Latini R., Libby P., Lombardi F., O’Gara P.T. (2012). Fish oil and postoperative atrial fibrillation: The omega-3 fatty acids for prevention of post-operative atrial fibrillation (OPERA) randomized trial. JAMA.

[130] Wilbring M., Ploetze K., Bormann S., Waldow T., Matschke K. (2014). Omega-3 Polyunsaturated Fatty Acids Reduce the Incidence of Postoperative Atrial Fibrillation in Patients with History of Prior Myocardial Infarction Undergoing Isolated Coronary Artery Bypass Grafting. Thorac. Cardiovasc. Surg..

[131] Saravanan P., West A.L., Bridgewater B., Davidson N.C., Calder P.C., Dobrzynsky H., Trafford A., O’Neill S.C. (2016). Omega-3 fatty acids do not alter P-wave parameters in electrocardiogram or expression of atrial connexins in patients undergoing coronary artery bypass surgery. Europace.

[132] Metcalf R.G., Skuladottir G.V., Indridason O.S., Sullivan T.R., Bjorgvinsdottir L., Sanders P., Arnar D.O., Gibson R.A., Heidarsdottir R., Cleland L.G. (2014). U-shaped relationship between tissue docosahexaenoic acid and atrial fibrillation following cardiac surgery. Eur. J. Clin. Nutr..

[133] Bjorgvinsdottir L., Arnar D.O., Indridason O.S., Heidarsdottir R., Skogstrand K., Torfason B., Hougaard D.M., Palsson R., Skuladottir G.V. (2013). Do high levels of n-3 polyunsaturated fatty acids in cell membranes increase the risk of postoperative atrial fibrillation?. Cardiology.

[134] Kumar S., Kassotis J.T. (2014). Do omega-3 polyunsaturated fatty acids prevent atrial fibrillation?. Cardiology.

[135] Armaganijan L., Lopes R.D., Healey J.S., Piccini J.P., Nair G.M., Morillo C.A. (2011). Do omega-3 fatty acids prevent atrial fibrillation after open heart surgery? A meta-analysis of randomized controlled trials. Clinics.

[136] Benedetto U., Angeloni E., Melina G., Danesi T.H., Di Bartolomeo R., Lechiancole A., Refice S., Roscitano A., Comito C., Sinatra R. (2013). N-3 Polyunsaturated fatty acids for the prevention of postoperative atrial fibrillation: A meta-analysis of randomized controlled trials. J. Cardiovasc. Med..

[137] Mozaffarian D., Wu J.H.Y., De Oliveira Otto M.C., Sandesara C.M., Metcalf R.G., Latini R., Libby P., Lombardi F., O’Gara P.T., Page R.L. (2013). Fish oil and post-operative atrial fibrillation: A meta-analysis of randomized controlled trials. J. Am. Coll. Cardiol..

[138] Mariani J., Doval H.C., Nul D., Varini S., Grancelli H., Ferrante D., Tognoni G., Macchia A. (2013). N-3 polyunsaturated fatty acids to prevent atrial fibrillation: Updated systematic review and meta-analysis of randomized controlled trials. J. Am. Heart Assoc..

[139] Ali-Hassan-Sayegh S., Mirhosseini S.J., Rezaeisadrabadi M., Dehghan H.R., Sedaghat-Hamedani F., Kayvanpour E., Popov A.-F., Liakopoulos O.J. (2014). Antioxidant supplementations for prevention of atrial fibrillation after cardiac surgery: An updated comprehensive systematic review and meta-analysis of 23 randomized controlled trials. Interact. Cardiovasc. Thorac. Surg..

[140] Costanzo S., Di Niro V., Di Castelnuovo A., Gianfagna F., Donati M.B., De Gaetano G., Iacoviello L. (2013). Prevention of postoperative atrial fibrillation in open heart surgery patients by preoperative supplementation of n-3 polyunsaturated fatty acids: An updated meta-analysis. J. Thorac. Cardiovasc. Surg..

[141] Zhang B., Zhen Y., Tao A., Bao Z., Zhang G. (2014). Polyunsaturated fatty acids for the prevention of atrial fibrillation after cardiac surgery: An updated meta-analysis of randomized controlled trials. J. Cardiol..

[142] Xin W., Wei W., Lin Z., Zhang X., Yang H., Zhang T., Li B., Mi S. (2013). Fish Oil and Atrial Fibrillation after Cardiac Surgery: A Meta-Analysis of Randomized Controlled Trials. PLoS ONE.

[143] Guo X.Y., Yan X.L., Chen Y.W., Tang R.B., Du X., Dong J.Z., Ma C.S. (2014). Omega-3 fatty acids for postoperative atrial fibrillation: Alone or in combination with antioxidant vitamins?. Heart Lung Circ..

[144] Christou G.A., Christou K.A., Korantzopoulos P., Rizos E.C., Nikas D.N., Goudevenos J.A. (2015). The current role of Omega-3 fatty acids in the management of atrial fibrillation. Int. J. Mol. Sci..

[145] Virtanen J.K., Mursu J., Voutilainen S., Tuomainen T.P. (2009). Serum long-chain n-3 polyunsaturated fatty acids and risk of hospital diagnosis of atrial fibrillation in men. Circulation.

[146] Ramadeen A., Connelly K.A., Leong-Poi H., Hu X., Fujii H., Laurent G., Domenichiello A.F., Bazinet R.P., Dorian P. (2012). Docosahexaenoic acid, but not eicosapentaenoic acid, supplementation reduces vulnerability to atrial fibrillation. Circ. Arrhythmia Electrophysiol..

[147] Wu J.H.Y., Lemaitre R.N., King I.B., Song X., Sacks F.M., Rimm E.B., Heckbert S.R., Siscovick D.S., Mozaffarian D. (2012). Association of plasma phospholipid long-chain omega-3 fatty acids with incident atrial fibrillation in older adults: The cardiovascular health study. Circulation.

[148] Kumar S., Sutherland F., Lee J.M.S., Robinson T., Heck P.M., Wong M.C.G., Kelland N.F., Garg M.L., Sparks P.B. (2013). Effects of high dose intravenous fish oil on human atrial electrophysiology: Implications for possible anti- and pro-arrhythmic mechanisms in atrial fibrillation. Int. J. Cardiol..

[149] Mozaffarian D., Rimm E.B. (2006). Fish Intake, Contaminants, and Human Health. JAMA.

[150] Tribulova N., Bacova B.S., Benova T.E., Knezl V., Barancik M., Slezak J. (2017). Omega-3 index and anti-arrhythmic potential of omega-3 PUFAs. Nutrients.

[151] Langlois P.L., Hardy G., Manzanares W. (2017). Omega-3 polyunsaturated fatty acids in cardiac surgery patients: An updated systematic review and meta-analysis. Clin. Nutr..

[152] Wang H., Chen J., Zhao L. (2018). N-3 polyunsaturated fatty acids for prevention of postoperative atrial fibrillation: Updated meta-analysis and systematic review. J. Interv. Card. Electrophysiol..

[153] Carnes C.A., Chung M.K., Nakayama T., Nakayama H., Baliga R.S., Piao S., Kanderian A., Pavia S., Hamlin R.L., McCarthy P.M. (2001). Ascorbate attenuates atrial pacing-induced peroxynitrite formation and electrical remodeling and decreases the incidence of postoperative atrial fibrillation. Circ. Res..

[154] Eslami M., Sattarzadeh Badkoubeh R., Mousavi M., Radmehr H., Salehi M., Tavakoli N., Avadi M.R. (2007). Oral ascorbic acid in combination with beta-blockers: Is more effective than beta-blockers alone in the prevention of atrial fibrillation after coronary artery bypass grafting. Texas Heart Inst. J..

[155] Papoulidis P., Ananiadou O., Chalvatzoulis E., Ampatzidou F., Koutsogiannidis C., Karaiskos T., Madesis A., Drossos G. (2011). The role of ascorbic acid in the prevention of atrial fibrillation after elective on-pump myocardial revascularization surgery: A single-center experience—A pilot study. Interact. Cardiovasc. Thorac. Surg..

[156] Samadikhah J., Golzari S.E.J., Sabermarouf B., Karimzadeh I., Tizro P., Khanli H.M., Ghabili K. (2014). Efficacy of combination therapy of statin and vitamin C in comparison with statin in the prevention of post-CABG atrial fibrillation. Adv. Pharm. Bull..

[157] Dehghani M.R., Madjidi N., Rahmani A., Asgari B., Rezaei Y. (2014). Effect of oral vitamin C on atrial fibrillation development after isolated coronary artery bypass grafting surgery: A prospective randomized clinical trial. Cardiol. J..

[158] Colby J.A., Chen W.T., Baker W.L., Coleman C.I., Reinhart K., Kluger J., White C.M. (2011). Effect of ascorbic acid on inflammatory markers after cardiothoracic surgery. Am. J. Health Pharm..

[159] Bjordahl P.M., Helmer S.D., Gosnell D.J., Wemmer G.E., O’Hara W.W., Milfeld D.J. (2012). Perioperative supplementation with ascorbic acid does not prevent atrial fibrillation in coronary artery bypass graft patients. Am. J. Surg..

[160] Sadeghpour A., Alizadehasl A., Kyavar M., Sadeghi T., Moludi J., Gholizadeh F., Totonchi Z., Ghadrdoost B. (2015). Impact of vitamin C supplementation on post-cardiac surgery ICU and hospital length of stay. Anesthesiol. Pain Med..

[161] Antonic M., Lipovec R., Gregorcic F., Juric P., Kosir G. (2017). Perioperative ascorbic acid supplementation does not reduce the incidence of postoperative atrial fibrillation in on-pump coronary artery bypass graft patients. J. Cardiol..

[162] Mirmohammadsadeghi M., Mirmohammadsadeghi A., Mahmoudian M. (2018). Preventive Use of Ascorbic Acid for Atrial Fibrillation After Coronary Artery Bypass Graft Surgery. Heart Surg. Forum.

[163] Hu X., Yuan L., Wang H., Li C., Cai J., Hu Y., Ma C. (2017). Efficacy and safety of vitamin C for atrial fibrillation after cardiac surgery: A meta-analysis with trial sequential analysis of randomized controlled trials. Int. J. Surg..

[164] Polymeropoulos E., Bagos P., Papadimitriou M., Rizos I., Patsouris E., Toumpoulis I. (2016). Vitamin C for the prevention of postoperative atrial fibrillation after cardiac surgery: A meta-analysis. Adv. Pharm. Bull..

[165] Geng J., Qian J., Si W., Cheng H., Ji F., Shen Z. (2017). The clinical benefits of perioperative antioxidant vitamin therapy in patients undergoing cardiac surgery: A meta-analysis. Interact. Cardiovasc. Thorac. Surg..

[166] Baker W.L., Coleman C.I. (2016). Meta-analysis of ascorbic acid for prevention of postoperative atrial fibrillation after cardiac surgery. Am. J. Health Syst. Pharm..

[167] Hemilä H. (2017). Publication bias in meta-analysis of ascorbic acid for postoperative atrial fibrillation. Am. J. Health Pharm..

[168] Van Wagoner D., Palumbo R., Li J., Carnes C., Gillinov A., McCarthy P., Chung M. Supplemental Vitamin C Did Not Reduce the Incidence of Atrial Arrhythmia following Cardiac Bypass Surgery. https://www.mv.helsinki.fi/home/hemila/CAF/vanWagoner2003.pdf.

[169] Donovan P., Kramer R. (2012). Prophylaxis to Reduce Postoperative Atrial Fibrillation in Cardiac Surgery.

[170] Healy R., Day D., van Gorder C. (2010). Ascorbic acid utilization for atrial-fibrillation prophylaxis post coronary-artery-bypass graft and valve replacement surgeries: An interim analysis of a prospective, randomized study. Pharmacotherapy.

[171] Hemilä H., Suonsyrjä T. (2017). Vitamin C for preventing atrial fibrillation in high risk patients: A systematic review and meta-analysis. BMC Cardiovasc. Disord..

[172] Hill A., Clasen K.C., Wendt S., Majoros G., Stoppe C., Adhikari N.K.J., Heyland D.K., Benstoem C. (2019). Effects of vitamin c on organ function in cardiac surgery patients: A systematic review and meta-analysis. Nutrients.

[173] Shi R., Li Z.H., Chen D., Wu Q.C., Zhou X.L., Tie H.T. (2018). Sole and combined vitamin C supplementation can prevent postoperative atrial fibrillation after cardiac surgery: A systematic review and meta-analysis of randomized controlled trials. Clin. Cardiol..

[174] Castillo R., Rodrigo R., Perez F., Cereceda M., Asenjo R., Zamorano J., Navarrete R., Villalabeitia E., Sanz J., Baeza C. (2011). Antioxidant Therapy Reduces Oxidative and Inflammatory Tissue Damage in Patients Subjected to Cardiac Surgery with Extracorporeal Circulation. Basic Clin. Pharmacol. Toxicol..

[175] Rodrigo R., Gutiérrez R., Fernández R., Guzmán P. (2012). Ageing improves the antioxidant response against postoperative atrial fibrillation: A randomized controlled trial. Interact. Cardiovasc. Thorac. Surg..

[176] Rodrigo R., Korantzopoulos P., Cereceda M., Asenjo R., Zamorano J., Villalabeitia E., Baeza C., Aguayo R., Castillo R., Carrasco R. (2013). A randomized controlled trial to prevent post-operative atrial fibrillation by antioxidant reinforcement. J. Am. Coll. Cardiol..

